# Comparison of maternal and neonatal serum leptin levels in preeclampsia and normal pregnancy

**Published:** 2011

**Authors:** Nesa Asnafi, Majid Sharbatdaran, Karimollah Hajian

**Affiliations:** 1 Department of Obstetrics and Gynecology, Babol University of Medical Sciences, Rohani Hospital, Babol, Iran.; 2 Department of Clinical Pathology, Babol University of Medical Sciences, Babol, Iran.; 3 Department of Social and Health Medicine, Babol University of Medical Sciences, Babol, Iran.

**Keywords:** *Leptin*, *Newborn*, *Pre-eclampsia*, *Umbilical cord*

## Abstract

**Background:** Leptin is a protein product of obesity gene and is synthesized mainly by adipose tissue.

**Objective:** The aim of this study was to determine maternal and neonatal serum leptin levels in term preeclamptic and normal pregnancies.

**Materials and Methods**: This cross sectional study was performed on 37 preeclamptic and 40 normotensive term pregnant women without other disease. Serum level of leptin was measured in all of pregnant mothers and after delivery, their neonates. This study was performed in Babol Yahyanejad Hospital from March 2006 to December 2006.

**Results:** Infants with preeclamptic mothers had significantly lower leptin level than control group (p=0.02). There was no significant difference in serum leptin levels between normal and preeclamptic women (p=0.749).

**Conclusion:** According to the results, it would be concluded that leptin level in infants of preeclamptic mothers is lower than infants of normal mothers. This can only confirm the diagnosis of disease after birth but it cannot predict the preeclampsia.

## Introduction

Leptin, a 16-KD polypeptide and the protein product of obesity gene, is synthesized mainly by adipose tissue (1, 2). Its level rises as body fat mass increases and contributes to energy homeostasis (2, 3). This 167-amino-acided protein is also present in ovaries, decidua and possibly contributes to the reproductive function (1, 2). Leptin may be detected in the umbilical cord from the 18th gestational week and its level increases up to term (2). 

On the other hand, leptin levels are higher among women than men because women have a BMI (Body Mass Index) with relatively higher content of fat and serum leptin level is proportional to adiposity. Preeclampsia, a systemic disease characterized by hypertension and proteinuria after the 20th gestational week, is due to incomplete trophoblastic invasion (3, 4) and diffuse endothelial dysfunctions (5, 6). During early pregnancy, when cytotrophoblasts invade the endometrium, they produce leptin which is probably involved in cytotrophblastic invasion. As placenta produces leptin, its hypoxia, occurred in preeclampsia, is likely to enhance leptin production (1, 7). Yet still, results about leptin concentration in preeclamptic women and their infants are controversial (3, 7-12). Some studies have detected its increase in pre-eclamptic women (7, 8, 13-18). But in others, it is found to be unchanged (9, 19, 20). The aim of this study was to compare maternal and umbilical vein serum leptin level between normotensive pregnant women as control group and preeclamptic patients.

## Materials and methods

In this cross-sectional study, we recruited a total number of 77 pregnant women (37 preeclamptic healthy women as group A and 40 normotensive healthy women as control group (group B) from Babol Yahyanejad Hospital from March to December 2006. This sample size can detect on average difference of 2ng/ml on leptin level in maternal serum and umbilical cord serum, with 95% confidence level and 80% power of statistical test. Two groups under study were matched with respect to maternal age and gestational age. Preeclampsia was diagnosed by hypertension (BP≥140/90 mmHg) and a 24-hour urine protein ≥300mg or a2+ protein in a random urine specimen using dipstick. 

The exclusion criteria were; women with preterm labor and patients with other systemic diseases such as diabetes and chronic hypertension. There was no history of systemic disease in the healthy pregnant control group. Venous blood samples were taken from each woman immediately before delivery. All patients were fasting for 6-8 hours prior to delivery. 

A 10cm segment of cord was double-clamped after delivery and blood sample was taken from umbilical vein. Then serum was separated and serum leptin level was measured. We used human leptin assay kit (L-IBL Code no. 27127) Elisa method and the minimal detectable concentration of leptin by this assay was 0.19mg/ml. In addition other data including mother and neonatal weights were recorded in a questionnaire. 

**Statistical analysis**

Data was analyzed by SPSS software, Fisher’s exact and t-test. P-value of less than 0.05 was considered significant. The ethics committee of Babol University approved the study and all cases gave informed consent. In statistical analysis, we used regression model in order to adjust the effect of maternal weight on maternal and umbilical cord leptin difference between case and control. 

## Results

The average (±SD) age of the patients was 28± 4.0 and 27±4.2 years in group A and B, respectively. The average (±SD) gestational age was 38±1.5 and 39±1.5 weeks in group A and group B, respectively. There were no significant differences regarding maternal age, gestational age and birth weight between group A and B, whereas mothers’ weight (before delivery) was found to be significantly higher in group A (p=0.006) (Table I). Also maternal serum leptin levels were not significantly different in two groups but there was significant lower umbilical cord leptin concentration in neonates delivered from preeclamptic women (p=0.018) (Table II). Table III shows the unadjusted and adjusted regression coefficient for difference in mean of maternal leptin serum level and umbilical cord serum level between case and control. Unadjusted regression coefficient of umbilical cord was significant while after adjusting for maternal weight, the magnitude of coefficient slightly reduced but it did not reach to significant level. 

**Table I:** The mean (  SD) age and clinical characteristics of the preeclamptic and normal pregnant women

	* **Group A (n=37) (Mean SD)**	**Group B (n=40) (Mea( SD)**	**p-value**
Age (years)	284.0	274.2	0.70
Mothers weight (kg)	82 ± 16.4	72.7 ± 12.3	0.006
Birth weight (kg)	3.31 ± 0.59	3.21 ± 0.51	0.61

* **Group A:** Preeclamptic women

**Group B:** Control women

**Table II:** The mean (± S.D) maternal and umbilical cord serum leptin levels in group A and B.

	* **Group A (n=37)**	**Group B (n=40)**	**p-value**
Maternal serum Leptin (ng/ml)	5.3 ± 5.1	4.9 ± 4.4	0.74
Umblical cord serum leptin (ng/ml)	2.8 ± 2.9	4.4 ± 4	0.043

* **Group A:** Preeclamptic women

**Group B:** Control women

**Table III:** Unadjusted and adjusted mean difference of maternal and umbilical cord leptin using regression model

**Dependent variable**	**Unadjusted coefficient** **mean difference ± SE**	**p-value**	**Adjusted ** * ** coefficient mean difference ± SE**	**p-value**
Maternal leptin (ng/ml)	0.35±1.09	0.75	-0.31±1.13	0.78
Umbilical cord leptin (ng/ml)	-1.7 (±0.81)	0.04	-1.5±0.85	0.07

Mean difference±SE= Mean case – mean control ± Standard error.

* The effect of maternal weight as covariate was adjusted using regression model.

## Discussion

In this study, the cases and controls in two groups were almost similar in gestational and maternal age, but mother’s weight in group A was significantly higher than that in control group, which is acceptable due to the edema in pre-eclamptic women. On the other hand, even though the preeclamptic group had higher serum leptin concentrations, no significant difference was found in maternal serum leptin levels between group A and B. Similarly, Salomon *et al* has reported leptin concentrations in preeclamptic and control group (19) to have no significant difference with each other and also Martinez- Abundis *et al* reported the serum leptin concentration to be equal in preeclamptic and normotensive pregnant women (20), but many other authors such as Vitoratos *et al* suggested significant higher leptin concentrations in preeclamptic women (21, 22).

In the study by Vitoratos, evaluation of mother's serum leptin was at early third trimester whereas in the current study, this evaluation was at term pregnancy and before delivery. This fact can probably explain the different results. In our study, although there was a significant lower umbilical cord leptin concentration in the neonates of preeclamptic group compared to those in normotensive group, but in regression model we did not find any significant difference between two groups (Table III). Mc Carthy *et al* suggested that cord leptin level was not significantly different between preeclampsia and normal pregnancy (14), but Ronnaug *et al* reported that cord plasma leptin concentrations in neonates of preeclamptic group was higher (4).

Many studies reported that placenta has an effect on the elevation of leptin concentration in maternal serum and fetal circulation during pregnancy (23, 24) and Mise *et al* has suggested that placental leptin production is increased in cases of severe preeclampsia (25), therefore leptin secreted from placenta may be involved in the pathogenesis of preeclampsia. While in this study, no significant difference was found between the serum leptin concentrations in preeclamptic and normotensive groups, which may be due to the presence of several mild preeclamptic women in our patients group. Since lower serum leptin concentrations were detected in neonates of pre-eclamptic group, more extensive studies should be designed to determine the role of leptin in preeclampsia. 

## References

[1] Chan TF, Su JH, Chung YF, Hsu YH, Yeh YT, Jong SB (2003). Amniotic fluid and maternal serum Leptin levels in pregnant women who subsequently develop preeclampsia. Eur J Obstet Gynecol Repord Biol.

[2] Domali E, Messinis IE (2002). Leptin in pregnancy. J Matern Fetal Neonat Med.

[3] Laivuori H, Gallaher MJ, Collura L, Crombleholme WR, Markovic N, Rajakumar A (2006). Relationships between maternal plasma Leptin, Placental Leptin mRNA and protein in normal pregnancy, pre-eclampsia and intrauterine growth restriction without pre-eclampsia. Mol Hum Reprod.

[4] Odegard RA, Vatten LJ, Nilsen ST, Salvesen KA, Austgulen R (2002). Umbilical cord plasma Leptin is increased in preeclampsia. Am J Obstet Gynecol.

[5] Cuningham FG, McDonald PC, Gant NF ( 2005). Williams obstetrics.

[6] Baker PN, Kingdom JC (2004). Preeclampsia.

[7] Lepercq J, Cuerre-Millo M, Andre J, Cauzac M, Hauguel-de Meuzon S (2003). Leptin: a potential marker of placental insufficiency. Gynecol Obstet Invest.

[8] Lepercq J, Catalano P, Hauguel de Mouzon S (2007). Leptin in pregnancy, Facts questions and future. Gynecol Obstet Fertil.

[9] Laml T, Preyer O, Hartmann BW, Ruecklinger E, Soeregi G, Wagenbichler P (2001). Decreased maternal serum Leptin in pregnancies complicated by preeclampsia. J Soc Gynecol Investing.

[10] Kauma S, Takacs P, Scordalakes C, walsh S, Green K, Peng T (2002). Increased endothelial monocyte chemoattractant protein -1 and inter-leukin -8 in preeclampsia. Obstet Gynecol.

[11] Diaz E, Halhali A, Luna C, Diaz L, Avila E, Larrea F (2002). Newborn birth weight correlates with placental zinc, umbilical insulin– like growth factor I ,and Leptin levels in preeclampsia. Arch Med Res.

[12] Bartha JL, Romero-carmona R, Escobar-Liompart M, Comino-Delgado R (2001). The relationships between Leptin and inflammatory cytkines in women with pre-eclampsia. Br J Obstet Gynecol.

[13] Salvatores M, Gennarelli G, Menato G, Massobrio M (2006). Leptin as apossible marker of augmented metabolic risk during pregnancy. Minerva Ginecol.

[14] McCarthy JF, Misra DN, Roberts JM (1999). Maternal plasma Leptin is increased in preeclampsia and positively correlates with fetal cord concentration. Am J Obstet Gynecol.

[15] Adali E, Yildizhan R, Kolusari A, Kurdoglu M, Bugdayci G, Sahin HG (2009). Increased visfatin and leptin in pregnancies complicated by pre-eclampsia. J Matern Fetal Neonatal Med.

[16] Iftikhar U, Iqbal A, Shakoor S (2010). Relationship between leptin and lipids during pre-eclampsia. J Pak Med Assoc.

[17] Sugathadasa BH, Tennekoon KH, Karunanayake EH, Kumarasiri JM, Wijesundere AP (2010). Association of -2548 G/A Polymorphism in the Leptin Gene with Preeclampsia/Pregnancy-induced Hypertension. Hypertens Pregnancy.

[18] Sucak A, Kanat-Pektas M, Gungor T, Mollamahmutoglu L (2010). Leptin levels and antihypertensive treatment in preeclampsia. Singapore Med J.

[19] Salomon LJ, Benattar C, Audibert F, Fernandez H, Duyme M, Taieb J (2003). Severe preeclampsia is associated with high inhibin a levels and normal Leptin levels 7 to 13 weeks into pregnancy. Am J Obstet Gynecol.

[20] Martinez-Abundis E, Gonzalez-Ortiz M, Pascoe-Gonzalez S (2000). Serum Leptin levels and the severity of preeclampsia. Arch Gynecol Obstet.

[21] Atamer Y, Kocyigit Y, Yokus B, Atamer A, Erden AC (2005). Lipid peroxidation, antioxidant defense, status of trace metals and leptin levels in preeclampsia. Eur J Obstet Gynecol Reprod Biol.

[22] Vitoratos N, Chrystodoulacos G, Kouskouni E, Salamalekis E, Creatsas G (2001). Alterations of maternal and fetal Leptin concentrations in hypertensive disorders of pregnancy. Eur J Obstet Gynecol Repord Biol.

[23] Hassink SG, de Lancey E, Sheslow DV, Smith-Kirwin SM, O'Connor DM, Considine RV (1997). Placental Leptin: an important new growth factor in intrauterine and neonatal development. Pediatrics.

[24] Yura S, Sagawa N, Mise H, Mori T, Masuzaki H, Ogawa Y (1998). A positive umbilical venous-arterial difference of Leptin level and its rapid decline after birth. Am J Obstet Gynecol.

[25] Mise H, Sagawa N, Matsumoto T, Yura S, Nanno H, Itoh H (1998). Augmented placental production of Leptin in pre-eclampsia possible involvement of placental hypoxia. J Clin Endoerinol Metab.

